# A Carboxyl Group-Functionalized Ionic Liquid Hybrid Adsorbent for Solid-Phase Extraction and Determination of Trace Diclofenac Sodium in Milk Samples

**DOI:** 10.3390/molecules28176216

**Published:** 2023-08-24

**Authors:** Hongrui Yang, Chen Wang, Wenjuan Zhu, Pingning Jin, Fei Li, Jing Fan

**Affiliations:** 1 School of Environment, Key Laboratory of Yellow River and Huai River Water Environment and Pollution Control, Ministry of Education, Henan Key Laboratory for Environmental Pollution Control, Henan Normal University, Xinxiang 453007, China; yangyangyang321200@163.com (H.Y.); wang2119084002@163.com (C.W.); wenjuanz87@126.com (W.Z.); jinpingning@126.com (P.J.); lifei40351991@163.com (F.L.); 2 College of Chemical and Environmental Engineering, Xinjiang Institute of Engineering, Urumqi 830023, China

**Keywords:** carboxyl group-functionalized ionic liquid, solid-phase extraction, diclofenac sodium, milk samples

## Abstract

A simple and efficient sample pretreatment technology is very important for the accurate determination of trace drug residues in foods to ensure food safety. Herein, we report a new carboxyl group-functionalized ionic liquid hybrid solid- phase adsorbent (PS-IL-COOH) for the highly efficient extraction and quantitative determination of diclofenac sodium (DS) residue in milk samples. It was found that the adsorption efficiency of PS-IL-COOH for the ppb level of DS was greater than 93.0%, the adsorption capacity was 934.1 mg/g, and the enrichment factor was 620.0, which surpass most of the previously reported values for DS adsorbents. The high concentration of salts did not interfere with the adsorption of DS. Importantly, the recovery of DS was above 90% after 16 adsorption–-regeneration cycles. The synergistic effect of the multiple interactions was found to be the main factor for the high efficiency of DS adsorption. The proposed method was applied to the extraction and detection of DS in milk samples, with the relative recovery ranging from 88.2 to 103.0%.

## 1. Introduction

Diclofenac sodium (DS) is a nonsteroidal anti-inflammatory drug that is widely used in humans, livestock, and fisheries [1] for the treatment of mammalian mastitis, arthritis, and sports injuries [2], because of its anti-inflammatory, antipyretic, and analgesic properties [3,4]. However, the overuse of DS may lead to unsafe residues in different animal tissues and animal-derived foods, posing a potential risk to consumers [5]. Even at low concentrations, long-term exposure can pose a major threat to human health, such as drug resistance, gastric ulcers, the inhibition of platelet aggregation, and an increased risk of heart attack [4]. Currently, residues of DS have been found in environmental water [6], the muscles of fish and shrimp [7], the livers of cattle and sheep [8], chicken wings, and milk [2,9]. Therefore, developing effective techniques for the accurate determination of trace DS in food samples is of great importance to ensure food safety.

As a drug residue, samples involving DS have the characteristics of complex composition and low content. In order to ensure the reliability of the determination results, sample pretreatment is an essential step. Currently, several methods have been reported for the pretreatment of samples involving DS. Among them, solid-phase extraction has attracted remarkable attention due to its simple operation, low usage amount of organic solvent, and high recovery efficiency [10,11,12]. In this context, Soheili-Azad et al. [13] reported a zinc/aluminum layered double hydroxide adsorbent (Zn/Al LDH) for the extraction and HPLC determination of trace DS from human serum and hospital wastewater. The DS adsorbed on the Zn/Al LDH was then eluted off with a 2.0 mol/L NaOH solution, and the enrichment factors were 37 (human serum) and 41 (hospital wastewater). Prasetya et al. [14] prepared a hollow fiber of a MOF-808 adsorbent for extracting DS from wastewater; the adsorption equilibrium time was 300 min with an adsorption capacity of up to 833 mg/g, and the adsorbent could withstand four adsorption–regeneration cycles with an eluent of methanol and acetic acid mixture (V:V = 9:1). Gao et al. [7] reported on a UIO-66-NH_2_ modified cotton fiber to extract DS from fish and shrimp muscle tissues. Coupled with ultra-high performance liquid chromatography detection, the detection limit was 0.12 ng/mL, and the enrichment factor was 32 with a 2% formic acid and methanol mixture (V:V = 1:1) as the eluent. The performance of the solid-phase extraction material is the key factor affecting the extraction efficiency [15]. Therefore, the design and synthesis of new, functionalized adsorbent materials with a short adsorption equilibrium time, large adsorption capacity, high enrichment factor, and green eluent are still the focus of researchers.

Ionic liquids (ILs) have attracted remarkable interest due to their extraordinary properties, such as low volatility, strong solubility, and controllable structure [16]. Therefore, they are considered to be satisfactory substitutes for traditional volatile organic solvents [17], and have been successfully applied to extraction [18], material preparation [19], organic synthesis [20], and other fields [21]. However, ILs are expensive, hard to regenerate, and easy to lose during use, which greatly limits their practical applications. In order to solve these problems, supported ionic liquid materials have attracted increasing attention because they have the unique properties of ionic liquids in solid supports [22].

In this work, a carboxyl group-functionalized ionic liquid (IL) hybrid solid adsorbent (which is named PS-IL-COOH because it has the properties of the carrier, and is supported by the functional group -COOH and the ionic liquid) is designed and synthesized in one step by chemically grafting N, N-dimethylglycine onto the surface of chloromethyl polystyrene (PS-Cl). It is found that the hybrid adsorbent can be used for the highly efficient separation and enrichment of DS in milk samples. Combined with a simple UV-Vis spectrophotometry, DS at the level of ppb can be directly detected. Moreover, a high concentration of inorganic salts does not affect the adsorption of trace amounts of the targeted drug, which improves the sensitivity and selectivity of the analytical method. In addition, the as-prepared adsorbent also has many advantages, such as a large adsorption capacity, high selectivity, fast adsorption speed, highly efficient regeneration and recycling performance, no need for expensive instruments, and almost no waste production in the whole sample pretreatment. This established separation and analysis method provides a new strategy for the green pretreatment of food samples and DS detection.

## 2. Results and Discussion

### 2.1. Characterization of PS-IL-COOH

**Figure 1:**
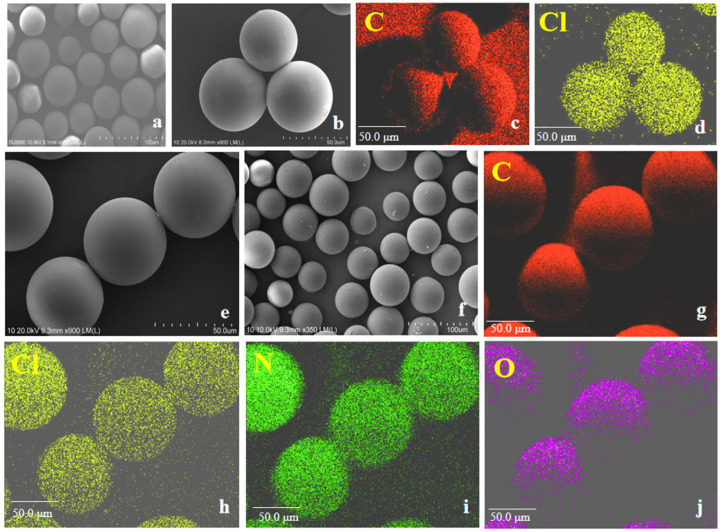
SEM images of PS-CH_2_Cl (**a**,**b**) and PS-IL-COOH (**e**,**f**), EDS mapping of PS-CH_2_Cl (**c**,**d**) and PS-IL-COOH (**g**–**j**).

The morphology of PS-CH_2_Cl and PS-IL-COOH are shown in Figure 1a,b,e,f. It is obvious that there are no obvious differences in the morphology between PS-CH_2_Cl and PS-IL-COOH. It is also clear that the new characteristic elements of nitrogen and oxygen appear in PS-IL-COOH (Figure 1i,j), which are not displayed in PS-CH_2_Cl, indicating that N, N-dimethylglycine successfully bonded onto the surface of the carrier.

Furthermore, as shown in Figure S1A, PS-CH_2_Cl remains almost unchanged in weight below 300 °C and gradually decomposes as the temperature rises; a weight loss of ~77% is observed from 300 to 550 °C. In the range of 100–200 °C, the slight weight loss of PS-IL-COOH is due to the evaporation of water, which could be attributed to the hydrophilic structure of the N, N-dimethylglycine that promotes the adsorption of a small amount of water by PS-IL-COOH in air. These results indicate that PS-IL-COOH has good thermal stability below 200 °C. According to the difference between the two thermogravimetric curves in Figure S1A, the grafting percentage of the IL in PS-IL-COOH is calculated to be approximately 17%, demonstrating that N, N-dimethylglycine was successfully bonded onto the PS-CH_2_Cl carrier.

**Scheme 1:**
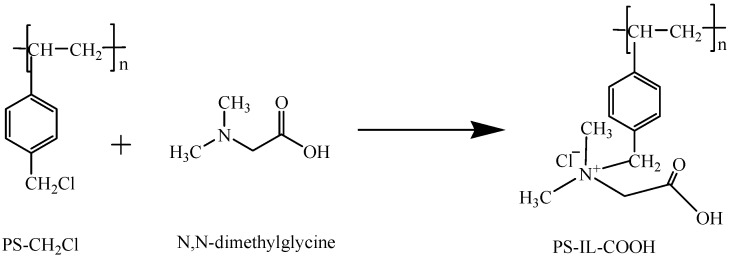
Preparation of PS-IL-COOH.

Next, the FT-IR spectra in Figure S1B show that the peaks at 1264 cm^−1^ and 671 cm^−1^ disappear in PS-IL-COOH, which is attributed to the C-Cl bending vibration and stretching vibration of PS-CH_2_Cl, respectively. However, a new characteristic peak of PS-IL-COOH appears at 1633 cm^−1^ (the COOH bending vibration) [23,24,25], suggesting that the N, N-dimethylglycine group was successfully grafted onto the surface of the carrier (Scheme 1). On the surface of the PS-CH_2_Cl carrier, there is the structure of an ionic liquid composed of carboxyl-functionalized cation and chloride anion. The IL on the surface of PS-IL-COOH can provide more active sites, give full play to the advantages of ionic liquids and, thus, significantly improve its adsorption capacity.

### 2.2. Optimization of Adsorption Performance

#### 2.2.1. Effects of pH and Temperature

**Figure 2:**
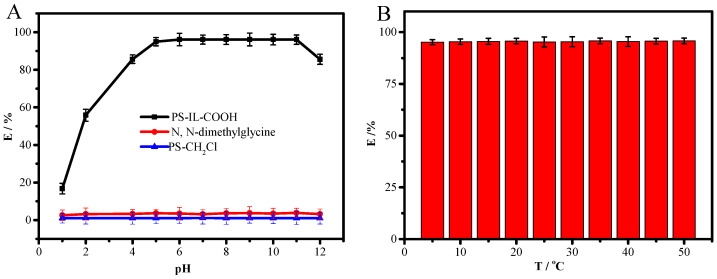
Effects of initial pH (**A**) and temperature (**B**) on the extraction efficiency of DS by PS-IL-COOH. Experimental conditions: m_PS-IL-COOH_ = 10 mg; C_DS_ = 20 μg/mL; V_DS_ = 5 mL; time = 30 min; room temperature in (**A**); and pH ≈ 6 in (**B**).

The pH value would affect not only the existing form of the target analyte but also the charge type of the adsorbent. Here, the effect of pH was investigated from 1 to 12. As shown in Figure 2A, it was found that PS-CH_2_Cl and N, N-dimethylglycine hardly adsorbed DS. However, for the as-prepared PS-IL-COOH adsorbent, the adsorption performance was enhanced significantly under a pH range from 1 to 5, and the adsorption capacity was at a maximum in the pH range of 5–11. This could be attributed to the fact that diclofenac (pKa = 4.15) mostly exists as neutral molecules in water in the range of pH < pKa [14]. When pH > pKa, DS mainly exists in the form of anions, as indicated by the positive zeta potential of PS-IL-COOH (Figure S2), and there is an electrostatic interaction between DS and PS-IL-COOH, which is favorable for improving the adsorption performance. All these points indicate that PS-IL-COOH had a good adsorption performance for DS in a wide range of pH. In order to avoid adjusting the pH of the sample solution, the following tests were carried out at a natural pH (pH ≈ 6) value.

The extraction efficiency of PS-IL-COOH towards DS was studied for a temperature range from 5 to 50 °C (Figure 2B). Clearly, temperature has little effect on the extraction efficiency, demonstrating that PS-IL-COOH can be applied at different temperatures without decreasing its adsorption performance. Therefore, room temperature was chosen for the subsequent experiments.

#### 2.2.2. Adsorption Capacity

**Figure 3:**
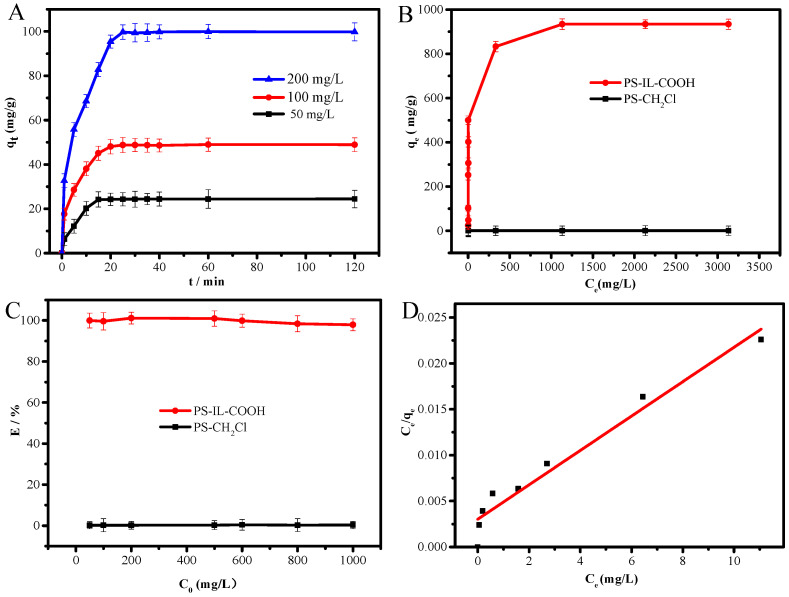
Adsorption capacity as a function of initial DS concentration. (**A**) The adsorption isotherm of PS-IL-COOH and PS-CH_2_Cl, (**B**) the extraction efficiency of PS-IL-COOH and PS-CH_2_Cl, and (**C**) the Langmuir model for the extraction of DS by PS-IL-COOH (**D**). Experimental conditions: m_PS-IL-COOH_ = 10 mg; time = 120 min; V_DS_ = 5 mL; pH ≈ 6; room temperature.

The adsorption capacity and adsorption equilibrium time are important parameters for assessing the adsorption performance of an adsorbent. As shown in Figure 3A, the adsorption capacity increases quickly with time in the initial absorption stage, and the adsorption equilibrium time is extended with an increase in DS initial concentration. This result can be ascribed to the abundant active sites of PS-IL-COOH in the initial stage. As these active adsorption sites are occupied, the speed of adsorption slows down, and the adsorption equilibrium is achieved in 15–25 min. As shown in Figure 3B, there is a significant rise in the equilibrium adsorption capacity of DS when the equilibrium concentration is smaller than 500 mg/L, then it stabilizes until adsorption reaches saturation with a maximum equilibrium adsorption capacity of 934.1 mg/g. In addition, the extraction efficiency of DS by PS-IL-COOH is 99.8–97.9% at an initial DS concentration range of 50–1000 mg/L, while the PS-CH_2_Cl carrier hardly adsorbs DS (Figure 3C), demonstrating that PS-IL-COOH is an excellent adsorbent for the extraction of DS.

To study the interaction between DS and PS-IL-COOH, the experimental data were fitted using various adsorption isotherm models (Table S1). The results are shown in Table S2. As shown in Figure 3D, the Langmuir model fitted the data best, and the maximum equilibrium adsorption capacity (965.3 mg/g) estimated using the Langmuir model is very close to the experimental value (934.1 mg/g).

#### 2.2.3. Extraction of Diclofenac Sodium at the Ppb Level

Because of the low concentration of residual DS in the actual samples, the ability of PS-IL-COOH to extract low concentrations of DS was estimated. An amount of 1.0 mL, 5 μg/mL solution of DS was diluted into a series of solutions in the concentration range of 0.0025–0.10 μg/mL, and then flowed through a column containing 100 mg of PS-IL-COOH at the optimized flow speed of 6 mL/min. Finally, the type and volume of eluent were investigated. As shown in Table 1, among aqueous HCl, NaOH, C_2_H_5_OH, and their mixtures, 3 mL of HCl-C_2_H_5_OH (V:V = 1:9) was the best eluent, with an elution recovery of 94.8%. Even when the concentration of DS was lower than 2.5 ppb, the elution recovery still reached 93.0%, with an enrichment factor (EF) as high as 620.0 (Table S3), as calculated using the equation EF = C*_f_*/C*_i_* [26], where C*_f_* and C*_i_* are the concentration of eluent and initial sample solution, respectively. All this suggests that PS-IL-COOH is a promising material for the extraction of DS at the ppb level.

#### 2.2.4. Selectivity of the Proposed Extraction Strategy

Coexisting substances in actual samples may affect the extraction and enrichment of DS. Thus, the influence of eight kinds of inorganic ions and seven kinds of organic compounds on the extraction efficiency of DS were studied in detail. For this purpose, different concentrations of the interfering substances were added to a solution of DS at a concentration of 10 μg/mL. After pretreatment with the proposed extraction strategy, the absorbance of DS in the eluent before and after adding the interfering substances was determined. It was considered that the concentration did not interfere with the extraction of DS when the relative standard deviation (RSD) was not greater than ±5%. As can be seen from Table 2, less interference is present from the inorganic ions; even when their concentrations were 1000 times that of DS, they still did not affect the extraction efficiency of PS-IL-COOH towards DS. The effect of divalent ions are greater than those of monovalent ions, supporting the viewpoint that electrostatic interaction is the main factor for the extraction of DS. However, the interference of the organic compounds is larger than that of the inorganic ions, and organic acid anions are more interferential than neutral molecules, suggesting that there may be hydrogen bonding in addition to the electrostatic interaction during the adsorption process. All this demonstrates that PS-IL-COOH has a potential application for the extraction and enrichment of DS from actual samples.

#### 2.2.5. Reusability of PS-IL-COOH

**Figure 4:**
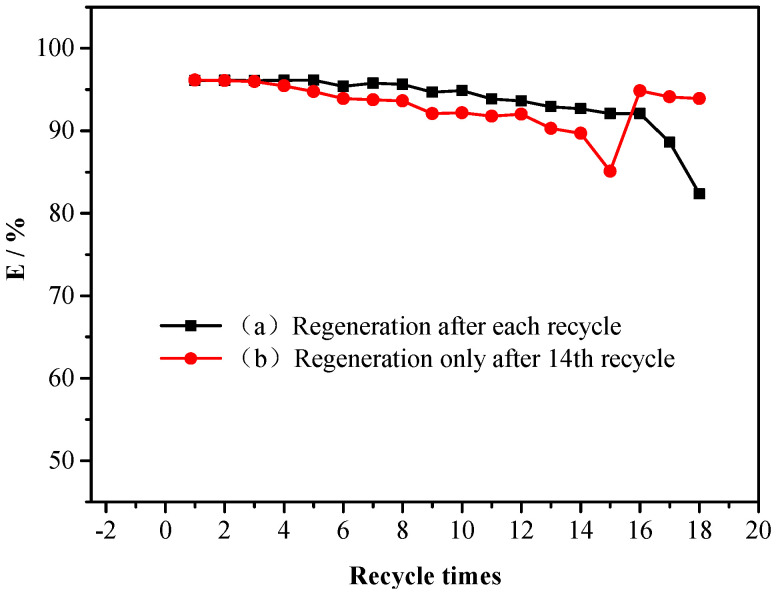
Influence of the recycling number of PS-IL-COOH on the extraction efficiency of DS: regeneration after each cycle (a) and regeneration only after the 14th cycle (b).

Regeneration and reuse is one of the most important properties for assessing the actual application of adsorbents [27]. Meanwhile, no waste discharge during sample pretreatment is also a goal of sustainable green sample processing. From the above discussion in Section 2.2.3, HCl-C_2_H_5_OH (V:V = 1:9) was used to regenerate PS-IL-COOH and recover DS. It was found that the elution recovery of DS was still above 90% even after 16 adsorption–-desorption cycles (Figure 4). In addition, PS-IL-COOH could be reused until adsorption saturation was reached. On the other hand, it is clearly seen from Figure 4 that PS-IL-COOH could maintain a high extraction efficiency in the first 14 adsorption cycles without regeneration, but exhibited a significant reduction in adsorption efficiency in the 15th cycle. However, after regeneration by using the eluent, the adsorption capacity of PS-IL-COOH recovered in the 16th cycle. Compared to the first strategy for the regeneration of PS-IL-COOH (regeneration after each recycle), the second strategy (regeneration after the 14th recycle) can greatly shorten the elution and sample- processing time, simplify the operation process, and reduce the amount of elution agent, which has a great actual application value for the recovery of DS. In addition, there was no clear difference in the UV-Vis adsorption of the recovered DS and the original DS under the same conditions (Figure S3), which demonstrates that the recovered DS has high purity. Simultaneously, as shown in Figure S4, there is little variation in the FT-IR spectrum between the regenerated PS-IL-COOH and fresh PS-IL-COOH, suggesting that the structure of the regenerated adsorbent was not destroyed, and the adsorption performance remained good. These results indicate that PS-IL-COOH is a potential material for the extraction of DS in actual samples.

#### 2.2.6. Method Validation

In order to evaluate the applicability of the proposed method, the limit of detection (LOD, S/N = 3) and limit of quantification (LOQ, S/N = 10) were determined. The precision was assessed using the relative standard deviations of inter-day (*n* = 6) and intra-day (*n* = 6), and the accuracy was validated by the average recovery. The results given in Table S4 indicate that the proposed method has the potential to detect DS in milk samples.

#### 2.2.7. Real Sample Analysis

**Figure 5:**
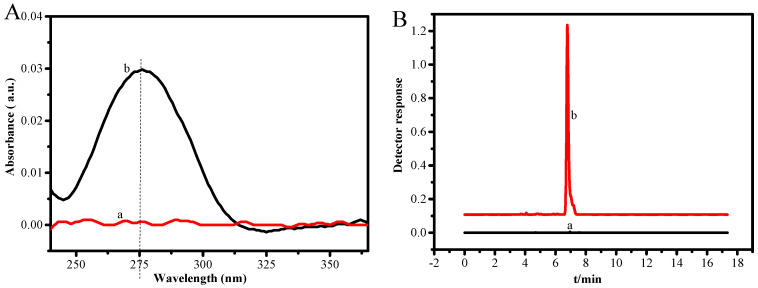
UV-Vis spectra of DS at 0.01 μg/mL before (a) and after (b) enrichment (**A**), and HPLC of DS at 0.01 μg/mL before (a) and after (b) enrichment (**B**). Experimental conditions of (**A**,**B**): pH ≈ 6; m_PS-IL-COOH_ = 100 mg; room temperature. Experimental conditions of (**B**): mobile phase= methanol-water (95:5, V:V); column temperature = 30 °C; sample volume = 20 μL; and detection wavelength = 280 nm.

Due to the complex matrix effects of food and environmental samples, most actual samples need to be pre-treated before instrumental analysis in order to remove matrix effects and enrich trace analytes. To verify the applicability of the established method, PS-IL-COOH was further used to separate and enrich different concentrations of DS (0.01, 1.0, and 10.0 μg/mL) in three milk samples. The milk samples were pretreated and determined by following the procedures described in Section 3.5 and Section 3.6. It is evident that the standard addition recovery is in the range of 88.2–103.0%, and the RSD is 2.5–7.3% (Table 3). The same 300 mL of DS (0.01 μg/mL) was flown through the column at 6 mL/min, and then the DS adsorbed on the PS-IL-COOH was eluted off with 3 mL of HCl-C_2_H_5_OH (V:V = 1:9) at 0.3 mL/min; the DS in the eluent was detected using UV-Vis spectrophotometry and HPLC (Figure 5A,B). It was clearly seen that the adsorbent had a good enrichment effect on trace DS, which was beneficial for the accurate determination of trace DS in actual samples.

To further evaluate the accuracy of the proposed method of sample pretreatment, the actual samples were separated and enriched with PS-IL-COOH; the determination results of UV-Vis spectrophotometry and HPLC were checked using the significance test (Table 4). It was found that there was no significant difference between UV-Vis and HPLC (*t* = 2.92 < t_critical_ = 4.30), indicating that UV-Vis spectrophotometry is reliable for the determination of trace DS in foods and environmental samples. From Figure 5A,B, it can be seen that the trace DS (0.01 μg/mL) could not be detected before enrichment because the instrument sensitivity was not high, while it could be detected successfully with UV-Vis spectrophotometry and HPLC after enrichment with PS-IL-COOH, with a recovery of 91.3% and 93.3%, respectively. This confirms that trace DS residues in milk samples pretreated with PS-IL-COOH can be accurately determined using UV-Vis spectrophotometry, which is simple, inexpensive, and does not consume large amounts of organic solvents, which is especially useful in economically undeveloped areas [28].

#### 2.2.8. Comparison with Other Adsorbents

To more clearly display the advantages of the PS-IL-COOH materials prepared in this study, the adsorption performance of PS-IL-COOH was compared to that of some previously reported adsorbents used for the adsorption of DS. It is shown in Table 5 that the PS-IL-COOH material has obvious advantages in terms of adsorption capacity, recycling number, and enrichment factor. Simultaneously, compared to commercially available adsorbents, PS-IL-COOH has obvious advantages in the extraction of DS both at low and high amounts under the same adsorption conditions (Table S5). The adsorption capacity of PS-IL-COOH is about 2.4–35.5 times that of commercially available adsorbents, indicating that PS-IL-COOH has significant potential for the extraction of DS from foods and environmental samples.

#### 2.2.9. Adsorption Mechanism

The study of the adsorption mechanism of pollutants is very beneficial to the development of an effective treatment technology. First, a comparison experiment was performed to study the main interactions between PS-IL-COOH and DS in an aqueous solution. We found that methyl orange (contains a typical organic anion) could be efficiently extracted, but rhodamine B (contains a typical organic cation) could not. Considering the fact that DS contains a bulk organic anion while PS-IL-COOH contains a bulk organic cation, this result suggests that electrostatic attraction plays an important role in the adsorption process, which was confirmed by the above mentioned pH effect of DS and the zeta potential of PS-IL-COOH.

In addition, it was found that after DS was adsorbed onto the surface of PS-IL-COOH (Figure S5), the stretching vibration peak of C=O of PS-IL-COOH (at 1633 cm^−1^) and the -N-H stretching vibration of DS (at 1310 cm^−1^) [35] were, respectively, shifted to 1630 cm^−1^ and 1315 cm^−1^. All these changes demonstrate that the -COOH on the surface of PS-IL-COOH may form a hydrogen bond with the N-H of DS.

**Figure 6:**
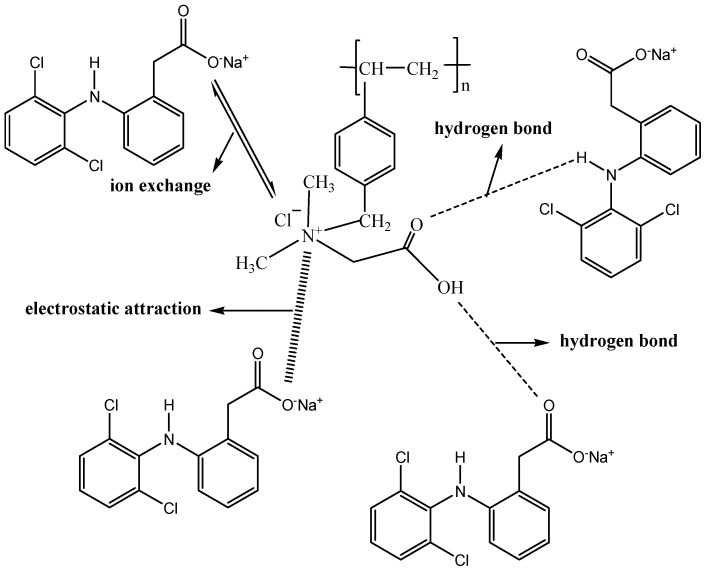
The possible adsorption mechanism of DS by PS-IL-COOH.

Another hypothesis is that there is an ion exchange between the anion of DS and the Cl- of PS-IL-COOH. To confirm this, 1 mL of supernatant was taken after the DS was adsorbed by the PS-IL-COOH, followed by adding one drop of HNO_3_ (0.1 mol/L) and then one drop of AgNO_3_ (0.1 mol/L), to see whether AgCl precipitate was produced. Subsequently, the result was compared to different blanks (Figure S6a–c) under the same conditions. It was found that AgCl precipitate appeared in the supernatant after the DS was adsorbed by PS-IL-COOH (Figure S6d), demonstrating that there was an ion exchange between the chloride ion of PS-IL-COOH and the anion of DS. And thus, the synergistic effect of electrostatic attraction, hydrogen bonding, and ion exchange plays a vital role in the super-high adsorption capacity of DS by PS-IL-COOH. The possible adsorption mechanism is shown in Figure 6.

## 3. Experimental Section

### 3.1. Reagents and Materials

Chloromethyl polystyrene resin (PS-CH_2_Cl, 200–400 mesh, chlorinity 3.5 mmol/g) was purchased from Tianjin Nankai Chemical Co. Ltd. (Tianjin, China). N, N-dimethylglycine (97%), N-methyl-2-pyrrolidone (98%), diclofenac sodium (99%), and trifluoroacetic acid (99%) were obtained from Aladdin Biochemical Technology Co., Ltd. (Shanghai, China). The other chemicals were all analytical grade.

### 3.2. Apparatus

A UV–Vis spectrophotometer (TU-1810, Beijing, China) was used for UV-Vis analysis of diclofenac sodium. The morphology and thermal stability of the materials were characterized using a Hitachi SU8010 scanning electron microscopy (Tokyo, Japan) and a Netzsch STA449C thermal analyzer (Selb, Germany), respectively. A FTS-40 Fourier-transform infrared spectroscopy (Waltham, MA, USA) was used to identify functional groups and interactions between materials and contaminants. A DC101-C oil bath (Zhengzhou, China) was used as the thermostat bath for synthesis purposes. A SPE-12B solid-phase extraction instrument (Tianjin, China) was used for the column adsorption experiments. The chromatographic analysis was performed using a waters 1525 HPLC system (Fleming, NY, USA), which was equipped with a 7725i manual sampler and 2998 ultraviolet detector.

### 3.3. Preparation of the Hybrid Solid-Phase Adsorbent

The carboxyl group-functionalized ionic liquid hybrid adsorbent (PS-IL-COOH) was synthesized using the following procedures: First, 0.5 g of PS-CH_2_Cl and 20 mL of N-methyl-2-pyrrolidone were added to a 50 mL round-bottomed flask and swelled the PS-CH_2_Cl for 12 h. Then, 1.084 g N, N-dimethylglycine was added with a speed of 700 rpm for a reaction time of 12 h at 80 °C in the oil bath. After the above mixtures were cooled to room temperature, they were transferred to a 50 mL centrifuge tube, and cleaned with deionized water and ethanol, respectively, and then centrifuged at 8000 rpm for 5 min. And then the supernatant was discarded, the mixture alternately cleaned with ethanol and deionized water until the N-methyl-2-pyrrolidone and the residue N, N-dimethylglycine were washed out. Finally, the target product was collected and then dried under vacuum at 60 °C for 12 h.

### 3.4. Preparation of the Solid-Phase Extraction Column Filled with PS-IL-COOH

The purchased polyethylene extraction column (10.0 mm in diameter and 62 mm in length) and several sieve pieces were soaked in ethanol for 24 h, cleaned with deionized water, and dried for use. A certain amount of PS-IL-COOH was weighed accurately and soaked in deionized water for 24 h as a backup. A sieve sheet was placed at the bottom of the extraction column, and the column was filled with deionized water. The column was loaded with the PS-IL-COOH using the overflow method, then a sieve sheet was placed on the column, and the PS-IL-COOH was compacted to make a miniature solid-phase extraction separation column for use.

### 3.5. Solid-Phase Extraction and Determination of Diclofenac Sodium

Adsorption experiments on the carboxyl group-functionalized ionic liquid hybrid adsorbent (PS-IL-COOH) towards diclofenac sodium (DS) were carried out using batch adsorption and column adsorption experiments. The batch adsorption experiment was performed at room temperature, where 5 mL sample of DS at different concentrations and 10 mg of PS-IL-COOH were added to a series of 20 mL sample bottles. After magnetic stirring for 30 min at 500 rpm to ensure the equilibrium of adsorption, the mixtures were transferred to a 10 mL centrifuge tube and then centrifuged at 8000 rpm for 5 min. A series of DS solutions with different concentrations were prepared with deionized water to make a standard curve, and then the concentration of the DS before and after adsorption could be determined using UV-Vis spectrophotometry at 275 nm. Finally, the concentration of DS adsorbed by the PS-IL-COOH could be quantitatively calculated according to the concentration difference before and after adsorption. The pH, temperature, adsorption equilibrium time, adsorption capacity, and anti-interference capability were investigated using batch experiments. The equilibrium adsorption capacity (q_e_) and the extraction efficiency (E%) of PS-IL-COOH towards DS were, respectively, calculated using Equations (1) and (2):

(1) $$q_{e}=\frac{\left(C_{0}-C_{e}\right)V}{m}$$

(2) $$E\%=\frac{\left(C_{0}-C_{e}\right)}{C_{0}}\times 100\%$$

where C_0_ (mg/L) and C_e_ (mg/L) are the concentrations of DS at the initial and the equilibrium (supernatant) concentration after adsorption, m (mg) is the mass of PS-IL-COOH, V (mL) is the volume of the solution of DS. In addition, we use q_t_ (mg/g) to present the absorption capacity at a time, t, and use R% to indicate the relative recovery of DS by PS-IL-COOH. All the experimental data were measured in triplicate.

The enrichment factor, real sample analysis, and regenerative capacity of the adsorbent were estimated with column adsorption experiments. Here, 100 mg of PS-IL-COOH was placed in a solid-phase extraction column and the DS solution was passed through the column at 6 mL/min. The DS adsorbed by the column was eluted with eluent at a flow speed of 0.3 mL/min, and then a series of DS solutions with different concentrations as reference eluents were used to make standard curves; the DS in the eluent was detected using UV-Vis spectrophotometry and confirmed using HPLC (mobile phase: methanol/water mixture (95: 5 = V:V), temperature of column: 30 °C, volume of sample: 20 μL, detection wavelength: 280 nm). The relative recovery (R%) was calculated according to Equation (3):

(3) $$R\%=\frac{q_{eluted}}{q_{added}}\times 100\%$$

where q_eluted_ and q_added_ are, respectively, the mass of eluted DS from the adsorbent and the added DS in the actual sample.

### 3.6. Real Sample Pretreatment and Analysis

The sample preparation procedure for three different brands of milk samples purchased at a local supermarket was based on a reported method [36]. A mixed solution of 60 mL of milk sample and 3 mL of trifluoroacetic acid (TFA) was centrifuged at 500 rpm for 5 min to separate the protein, the supernatant was filtered using a 0.22 μm membrane, and then the experiments of solid-phase extraction and determination were performed in accordance with Section 3.5.

## 4. Conclusions

In this work, a carboxyl group-functionalized ionic liquid hybrid adsorbent (PS-IL-COOH) was prepared using a one-step grafting reaction. The systematic studies show that PS-IL-COOH is a chemically stable solid phase adsorbent with an excellent adsorption performance for DS. The extraction efficiency still exceeded 93.0% even when the DS content was lower than 2.5 ppb. The enrichment factor and adsorption capacity reached up to 620.0 and 934.1 mg/g, respectively, which exceeds most of the previously reported values for DS adsorbents. In particular, PS-IL-COOH exhibited good salt resistance, and no effect was observed on the adsorption performance when the concentration of common inorganic salts was 1000–10,000 times that of DS. Moreover, under a wide range of pH and temperature, the recovery of DS was still above 90% after 16 adsorption–regeneration cycles, demonstrating its excellent environmental adaptability. These excellent adsorption properties may be attributed to the synergistic effects of electrostatic attraction, ion exchange, and hydrogen bonding of PS-IL-COOH with DS. All these results suggest that the adsorbent reported in the present work is outstanding and has great potential as an appealing sample pretreatment material to monitor trace pharmaceutical residuals in real food samples.

## Figures and Tables

**Table 1:** Elution recovery of DS in different eluents.

Eluent	R%	Eluent	R%
6 mol/L HCl	16.5	6 mol/L HCl-C_2_H_5_OH (5:5)	61.5
3 mol/L HCl	27.8	1 mol/L NaOH	13.3
C_2_H_5_OH	49.8	1 mol/L NaOH-C_2_H_5_OH (1:9)	88.7
6 mol/L HCl-C_2_H_5_OH (1:9)	94.8	1 mol/L NaOH-C_2_H_5_OH (2:8)	80.8
6 mol/L HCl-C_2_H_5_OH (2:8)	91.5	1 mol/L NaOH-C_2_H_5_OH (3:7)	73.1
6 mol/L HCl-C_2_H_5_OH (3:7)	80.0	1 mol/L NaOH-C_2_H_5_OH (4:6)	70.6
6 mol/L HCl-C_2_H_5_OH (4:6)	71.3	1 mol/L NaOH-C_2_H_5_OH (5:5)	67.1

Experimental conditions: m_PS-IL-COOH_ = 100 mg; pH ≈ 6; room temperature.

**Table 2:** The permissible interference ratio of common coexisting substances.

Coexisting Substances	Concentration (μg/mL)	Permit Ratio	RSD (%)
K^+^	100,000	10,000	1.6
Na^+^	100,000	10,000	2.3
Mg^2+^	10,000	1000	1.7
Ca^2+^	10,000	1000	1.5
Cl^−^	100,000	10,000	1.3
H_2_PO_4_^−^	100,000	10,000	1.9
CO_3_^2−^	10,000	1000	1.7
SO_4_^2−^	10,000	1000	2.4
glucose	18,000	1800	3.9
starch	3500	350	4.9
acetaminophen	4000	400	4.1
sulfanilamide	2000	200	2.6
citric acid	1000	100	1.9
L-alanine	1000	100	3.8
L-proline	1000	100	4.6

Experimental conditions: m_PS-IL-COOH_ = 10 mg; C_DS_ = 10 μg/mL; pH ≈ 6; room temperature.

**Table 3:** The recovery of PS-IL-COOH towards DS from milk samples.

Sample	Added Values (μg/mL)	Detected (μg/mL)	Recovery (%)	RSD (*n* = 3)
Milk sample 1	0.0	ND	ND	ND
	0.01	0.0091	91.0	2.9
	1.0	0.96	96.0	3.7
	10.0	9.41	94.1	5.2
Milk sample 2	0.0	ND	ND	ND
	0.01	0.0103	103.0	2.5
	1.0	1.02	102.0	3.4
	10.0	10.1	101.1	2.9
Milk sample 3	0.0	ND	ND	ND
	0.01	0.0098	98.0	3.1
	1.0	0.89	89.0	5.8
	10.0	8.82	88.2	7.3

ND: not detected.

**Table 4:** Comparison of the UV-Vis spectrophotometry and HPLC results.

Sample	Spiked (μg/mL)	UV-Vis Detected (μg/mL)	HPLC Detected (μg/mL)	*t*-Value ^a^
	0.01	0.0092	0.0091	
Milk sample 1	0.01	0.0089	0.0097	2.92
	0.01	0.0093	0.0092	

Experimental conditions: m_PS-IL-COOH_ = 100 mg; pH ≈ 6; room temperature. ^a^ paired *t*-test, 95% confidence level, *n* = 3, t_critical_ = 4.30.

**Table 5:** Comparison of the adsorption of DS by different adsorbents.

Adsorbent	q_e_	t_eq_	RN	EF	t_dt_	Ref.
CS@PANI@LDH composite	618.2	120	5	-	-	[29]
Fe_3_O_4_@MIL-100 (Fe)	400	60	5	-	-	[30]
Amine-functionalized egg albumin hydrogel	232.5	180	4	-	-	[31]
Bilayer amino-functionalized cellulose nanocrystals/chitosan composite	444.4	50	5	-	-	[32]
Amine-functionalized chitosan	469.5	60	4	-	-	[33]
Mg/Al layered double hydroxide-poly(m-phenylenediamine) composite	588	2	3	-	-	[34]
MOF-808 and its hollow fibre	833	300	4	-	-	[14]
Magnetic covalent organic frameworks	-	60	3	37.2	5	[15]
PS-IL-COOH	934.1	30	16	620.0	10	This work

q_e_: adsorption capacity (mg/g); t_eq_: equilibrium time (min); RN: recycling number; EF: enrichment factor; t_dt_: desorption time (min); t_et_: enrichment time (min), t_et_ = t_eq_ + t_dt_.

## Data Availability

This study presents novel concepts and did not report any data.

## Supplementary Materials

The following supporting information can be downloaded at: https://www.mdpi.com/article/10.3390/molecules28176216/s1. Table S1. The isotherm model and its equations; Table S2. The related parameters of various adsorption isotherm models; Table S3. The recovery and enrichment factor of PS-IL-COOH at different concentrations of DS; Table S4. Analytical performance of the proposed method on milk samples; Table S5. Comparison of the extraction of DS using PS-IL-COOH and commercial adsorbents; Figure S1. (A): The TGA curves of PS-CH_2_Cl (a) and PS-IL-COOH (b); (B): FT-TR spectra of PS-CH_2_Cl (a) and PS-IL-COOH (b); Figure S2. Zeta potential of PS-IL-COOH; Figure S3. UV-visible adsorption spectra of original DS (a) and the recovered DS (b); Figure S4. FT-IR spectra of the fresh PS-IL-COOH (a) and the regenerated PS-IL-COOH (b); Figure S5. FT-IR spectra of the PS-IL-COOH (a), PS-IL-COOH-DS (b), and DS (c); Figure S6. The photographs of different blank solutions and the adsorbed DS solution after addition of aqueous AgNO_3_: (a) deionized water, (b) aqueous DS solution, (c) supernatant after soaking of PS-IL-COOH, and (d) supernatant after DS was adsorbed by PS-IL-COOH. Experimental conditions: C_DS_ = 100 mg/L; V_DS_ = 5 mL; m_PS-IL-COOH_ = 10 mg; time = 30 min; pH ≈ 6; room temperature.

## Sample Availability

Samples of the compounds are available from the authors.
