# Supplementary material for: A Carboxyl Group-Functionalized Ionic Liquid Hybrid Adsorbent for Solid-Phase Extraction and Determination of Trace Diclofenac Sodium in Milk Samples
Source: Molecules. 2023 Aug 24;28(17):6216. doi: 10.3390/molecules28176216 (PMC10488911; doi:10.3390/molecules28176216)
Supplement: Supplementary file 1 [file molecules-28-06216-s001.zip › molecules-2545346-supplementary.pdf]

Supplementary material

A Carboxyl Group-functionalized Ionic Liquid Hybrid Adsorbent for Solid-phase Extraction and Determination of Trace Diclofenac Sodium in Milk Samples

Hongrui Yang[a, b], Chen Wang[a], Wenjuan Zhu[a], Pingning Jin[a], Fei Li[a], Jing Fan[a*]

[a] School of Environment, Key Laboratory of Yellow River and Huai River Water Environment and Pollution Control, Ministry of Education, Henan Key Laboratory for Environmental Pollution Control, Henan Normal University, Xinxiang, Henan 453007, PR China

[b] College of Chemical and Environmental Engineering, Xinjiang Institute of Engineering, Urumqi, 830023

Table of contents:

1. **Table S1.** The isotherm model and its equations.
2. **Table S2.** The related parameters of various adsorption isotherm models.
3. **Table S3.** The recovery and enrichment factor of PS-IL-COOH at different concentrations of DS.
4. **Table S4.** Analytical performance of the proposed method in milk sample.
5. **Table S5.** Comparison for the extraction of DS by PS-IL-COOH and commercial adsorbents.
6. **Figure S1.** (A): The TGA curves of PS-CH_2Cl (a) and PS-IL-COOH (b); (B): FT-TR spectra of PS-CH_2Cl (a) and PS-IL-COOH (b).
7. **Figure S2.** Zeta potential of PS-IL-COOH
8. **Figure S3.** UV-visible adsorption spectra of original DS (a) and the recovered DS (b).
9. **Figure S4.** FT-IR spectra of the fresh PS-IL-COOH (a) and the regenerated PS-IL-COOH (b).
10. **Figure S5.** FT-IR spectra of the PS-IL-COOH (a), PS-IL-COOH-DS (b), and DS (c).
11. **Figure S6.**The photographs for different blank solutions and the adsorbed DS solution after addition of aqueous $AgNO_3$: (a) deionized water, (b) aqueous DS solution, (c) supernatant after soaking of PS-IL-COOH, (d) supernatant after DS was adsorbed by PS-IL-COOH. Experimental conditions: C_{DS}=100 mg/L, V_{DS}=5 mL, $m_{PS\text{-}IL\text{-}COOH}$=10 mg, time=30 min, pH≈6, room temperature.

Table S1. The isotherm model and its equations.

Isotherm model	Equation	
Langmuir	$\frac{C_e}{q_e} = \frac{C_e}{q_m} + \frac{1}{q_m K_L}$	(1)
Freundlich	$Inq_e = InK_F + \frac{1}{n} InC_e$	(2)
Tempkin	$q_e = K_T InC_e + K_T Inf$	(3)

where q_e (mg/g) and q_m (mg/g) are, respectively, the equilibrium adsorption capacity and the maximum adsorption capacity. C_e (mg/L) stands for the equilibrium concentration of DS, K_L, K_F and K_T are the constant of Langmuir, Freundlich and Tempkin, respectively, and f is the maximum binding energy.

Table S2. The related parameters of various adsorption isotherm models.

Langmuir model			Freundlich model			Tempkin model		
K_L(L/mg)	q_m(mg/g)	R^2	K_F	n	R^2	K_T(L/mg)	f	R^2
5.15×10^{-4}	965.3	0.9509	0.148	1.71	0.8791	139.4	11.3	0.9264

Table S3. The recovery and enrichment factor of PS-IL-COOH at different concentrations of DS.

V_{DS} (mL)	C_i (μg/mL)	R%	C_f (μg/mL)	EF	RSD (%)
50.0	0.1	99.6	1.66	16.6	0.7
100.0	0.05	99.0	1.65	33.0	1.6
250.0	0.02	99.0	1.65	82.5	0.9
500.0	0.01	96.2	1.60	160.0	1.3
1000.0	0.005	94.2	1.57	314.0	1.2
2000.0	0.0025	93.0	1.55	620.0	2.1

Experimental conditions: $m_{PS\text{-}IL\text{-}COOH}$= 100 mg, pH≈6, room temperature.

Table S4. Analytical performance of the proposed method in milk sample.

**Parameter**	**Obtained values**
Calibration curve	y=0.03056x+0.00231
R^2	0.9996
LOD (μg/mL)	0.003
LOQ (μg/mL)	0.01
Inter-day RSD, %	3.7
Intra-day RSD, %	2.9
Average Recovery, %	93±3

Table S5. Comparison for the extraction of DS by PS-IL-COOH and commercial adsorbents.

Adsorbent	$C_{initial\ DS}$=10 mg/L		$C_{initial\ DS}$=1000 mg/L	
	E%	q_e (mg/g)	E%	q_e (mg/g)
Activated carbon	44.0	4.40	39.71	397.08
Activated alumina	12.4	1.24	11.72	117.21
Silica gel	12.0	1.20	10.94	109.39
Artificial zeolite	4.70	0.47	2.63	26.33
Weak-base anion exchange	43.1	4.31	36.51	365.1
PS-IL-COOH (this work)	100	10	93.41	934.12

Experimental conditions: $m_{adsorbent}$=10 mg, $V_{initial\ DS}$=10 mL, t=30 min, pH≈6, room temperature.

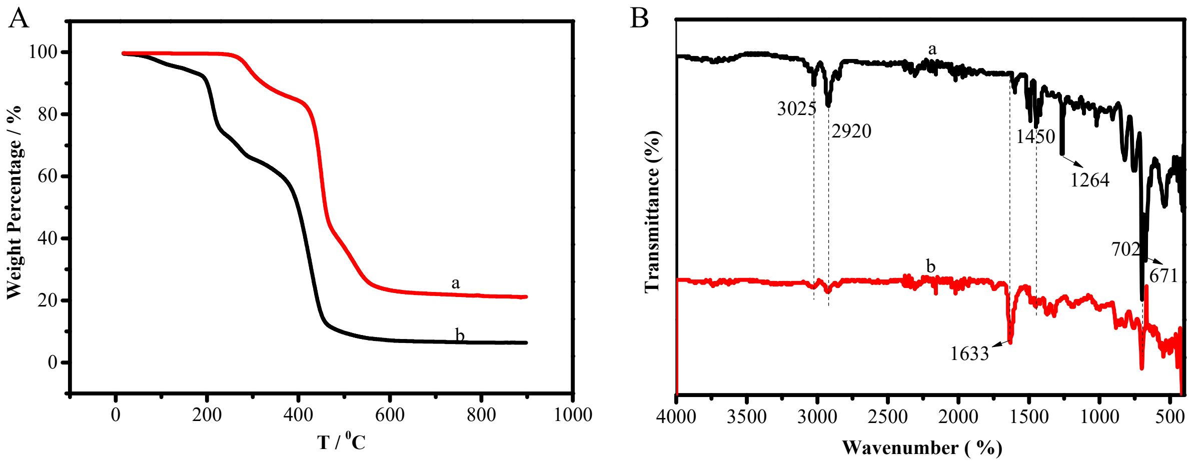

Figure S1. (A) The TGA curves of PS-CH_2Cl (a), and PS-IL-COOH (b); (B) FT-TR spectra of PS-CH_2Cl (a) and PS-IL-COOH (b).

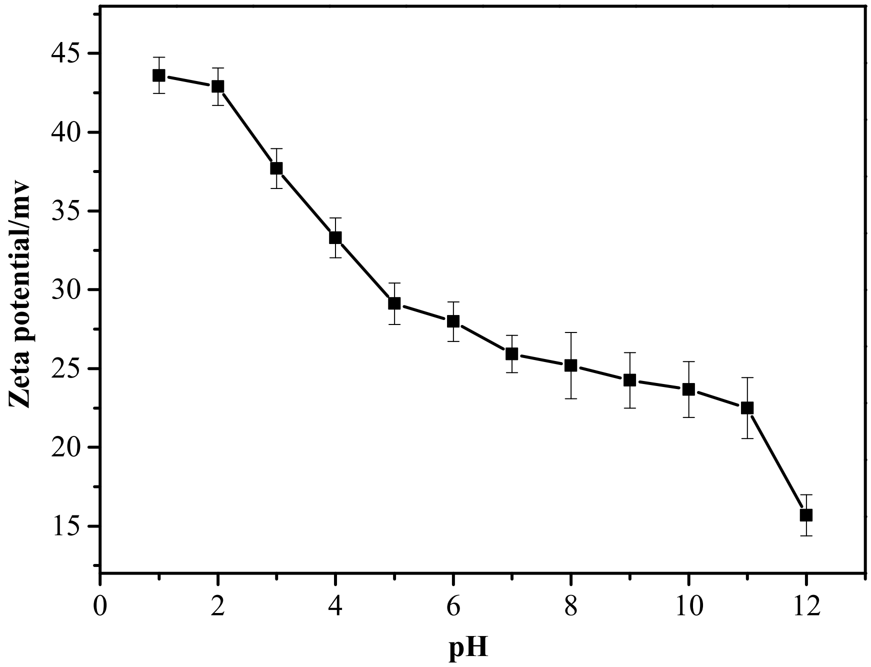

Figure S2. Zeta potential of PS-IL-COOH

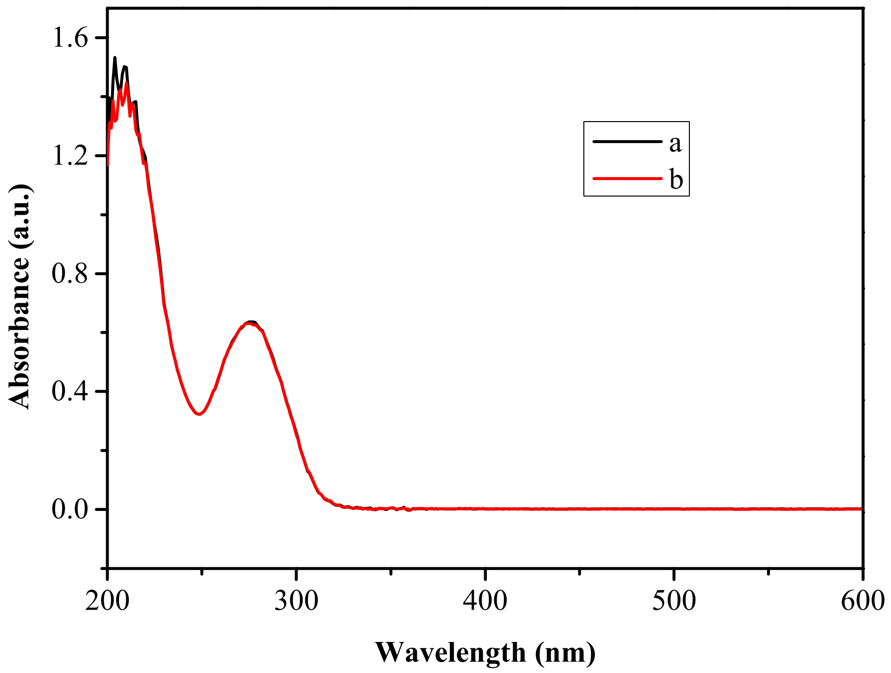

Figure S3. UV-visible adsorption spectra of the original DS (a) and the recovered DS (b).

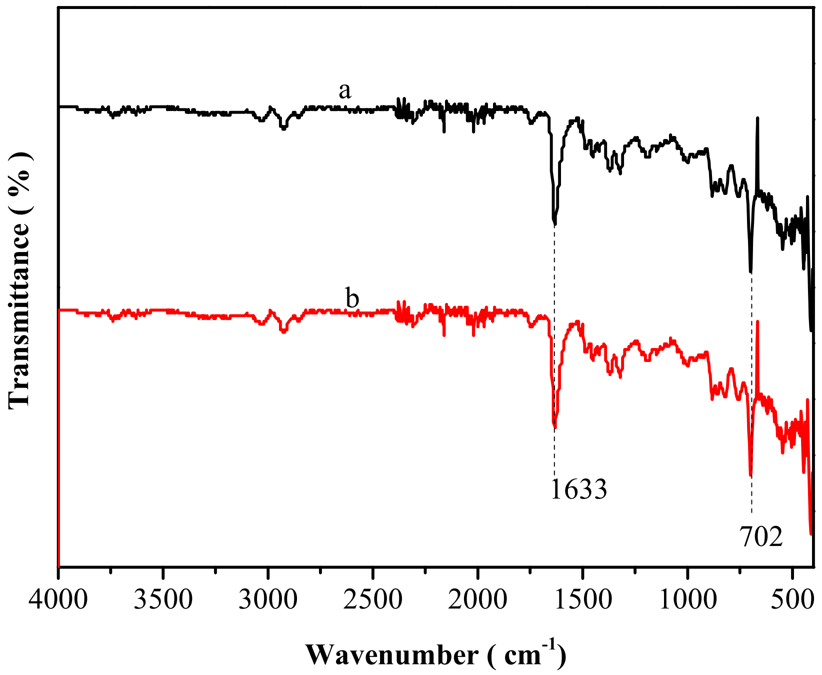

Figure S4. FT-IR spectra of the fresh PS-IL-COOH (a) and the regenerated PS-IL-COOH (b).

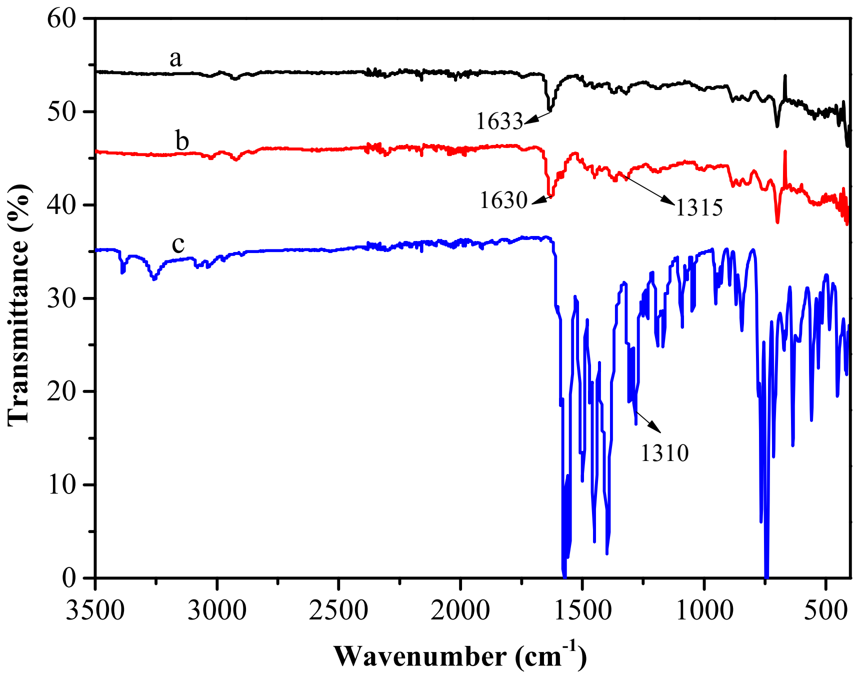

Figure S5. FT-IR spectra of the PS-IL-COOH (a), PS-IL-COOH-DS (b), and DS (c).

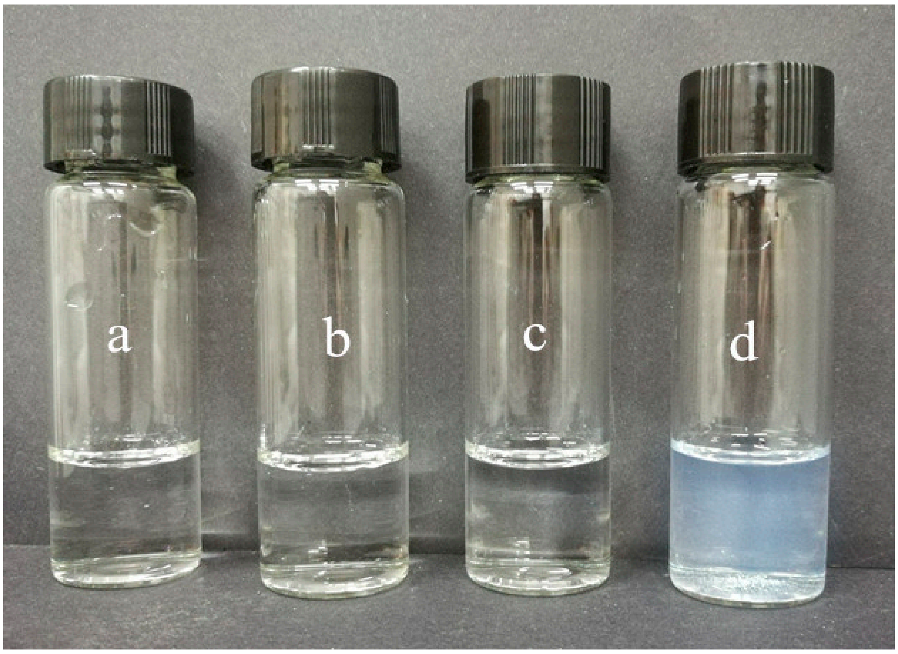

Figure S6. The photographs for different blank solutions and the adsorbed DS solution after addition of aqueous $AgNO_3$: (a) deionized water, (b) aqueous DS solution, (c) supernatant after soaking of PS-IL-COOH, (d) supernatant after DS was adsorbed by PS-IL-COOH. Experimental conditions: C_{DS}=100 mg/L, V_{DS}=5 mL, $m_{PS\text{-}IL\text{-}COOH}$=10 mg, time=30 min pH≈6, room temperature.
